# Plant-Based Diets and Climate Change, A Perspective for Infectious Disease Providers

**DOI:** 10.1093/ofid/ofaf222

**Published:** 2025-10-10

**Authors:** Melissa Whitman, Aldo Barajas-Ochoa, Sangeeta Sastry, Gonzalo Bearman

**Affiliations:** Division of Infectious Diseases, Department of Medicine, Virginia Commonwealth University, Richmond, Virginia, USA; Infectious Diseases, Asante Three Rivers Medical Center, Grants Pass, Oregon, USA; Division of Infectious Diseases, Department of Medicine, Virginia Commonwealth University, Richmond, Virginia, USA; Division of Infectious Diseases, Department of Medicine, Virginia Commonwealth University, Richmond, Virginia, USA

**Keywords:** climate change, dietary counseling, health outcomes, planetary health, plant-based diet

## Abstract

Global climate change driven by human activity is a pressing concern. Recent medical literature highlights the negative consequences of climate change on human health, including changing patterns and rising rates of global infectious diseases. Livestock production and animal agriculture are large contributors to greenhouse gas emissions, and rising rates of antimicrobial resistance are propagated by antibiotic use in livestock feed. Despite this, the global demand for animal-based food production continues to rapidly grow. Furthermore, meat consumption is linked to negative health consequences while plant-based diets provide health benefits that are endorsed by multiple medical associations as part of a healthy lifestyle. Health care providers, including infectious diseases physicians, are in a privileged position to provide dietary counseling. This review advocates for the adoption of plant-based diets as a dual strategy to combat climate change and improve health outcomes, particularly in the context of infectious diseases.

## Teaser

Global climate change driven by human activity is an urgent threat to human health and climate-sensitive infectious diseases. Plant-based diets are more environmentally sustainable than animal-based food products. Plant-based diets offer health benefits, and healthcare providers should consider dietary counseling as a part of patient care.

At current rates, global warming is estimated to reach 1.5°C between 2030 and 2052 compared with the pre-industrial era, in part due to human-related activities [1]. After fossil fuel emissions, deforestation and livestock are 2 of the greatest drivers of greenhouse gas emissions and thus climate change related to human activity. The United Nations estimates that a third of human-caused greenhouse gas emissions are due to food production, with 2 times higher emissions from animal products compared with plant-based foods [2, 3]. Deforestation and land use related specifically to animal food production cause multiple other negative consequences including biodiversity loss, pollution, and increased atmospheric carbon dioxide levels.

The medical community acknowledges climate change and its implications for human health. Multiple well-recognized journals emphasize its direct health consequences including heat-related illnesses [4] and increases in vector-borne diseases [5], particularly in vulnerable populations. In fact, about 60% of known infectious diseases were at some time aggravated by climatic hazards, events in part driven by the effects of climate change [6]. The World Health Organization's “One Health” concept recognizes this and acknowledges the relatedness of human health, animals, and their shared environment [7]. In addition to recognition, the medical community can help facilitate changes aimed at mitigating the devastating impacts of climate change on human health.

One way health care providers, including infectious diseases physicians, may play a role is through dietary counseling, with an emphasis on shifts toward a more environmentally favorable plant-based diet. Plant-based diets represent a continuum of diverse dietary patterns that consist mostly of foods of nonanimal origin including minimally processed fruits, vegetables, grains, nuts, and legumes (Table 1). Plant-based diets demonstrate numerous health benefits [8–10], with dietary changes that favor reduced consumption of animal products having a more positive environmental impact. This review aims to highlight the environmental and health benefits of a plant-based diet, followed by actionable steps for dietary counseling that can result in positive health consequences for both the individual and the environment.

**Table 1.** Types of Plant-Based Dietary Patterns

Type of Plant-Based Dietary Pattern	Red Meat	Fish and Seafood	Poultry	Eggs	Milk and Dairy Products	Vegetables, Nuts, Legumes, Grains, and Fruits
Flexitarian^a^	X	X	X	X	X	X
Pescatarian		X		X	X	X
Pollotarian/pollo-vegetarian			X	X	X	X
Lacto-ovo-vegetarian				X	X	X
Ovo-vegetarian				X		X
Lacto-vegetarian					X	X
Vegan						X

^a^Diet is composed mainly of plant-foods and occasionally includes animal-based foods.

## ENVIRONMENTAL IMPACT

The animal agriculture sector is a major source of greenhouse gas emissions. It is estimated that half of the world's habitable land is devoted to agriculture, with two-thirds of that utilized for animal livestock [11, 12]. The meat industry is responsible for almost half of global methane emissions, with a single cow producing around 200 pounds of methane gas annually [13]. Animal agriculture is additionally a significant contributor to deforestation and biodiversity loss [14, 15]. In 2023, 28 million hectares of tree cover (about the size of the United Kingdom) were lost to deforestation alone [16]. Land use as pasture accounted for 36% of all tree loss due to agriculture between 2001 and 2015, with cattle responsible for 2 times higher forest replacement than all other agricultural commodities combined [17]. Despite this, the industry continues to grow in response to heightened food demand, with meat consumption estimated to rise 76% by 2050 [18].

The consequences of the livestock industry are widespread and far-reaching. Deforestation and land clearing for agriculture result in habitat alteration by increasing the amount of standing water, extending breeding grounds for insect vectors [19]. The effect of microclimates in cleared lands compared with forested areas additionally promotes mosquito survival and fitness, with vectors carrying human pathogens more abundant in deforested than forested areas [19, 20]. Furthermore, deforestation directly leads to habitat destruction and decreases animal biodiversity. The consequences of this include inbreeding and decreased immune health, both of which can lead to increases in the transmission of zoonotic and vector-borne diseases [21, 22].

Transmission of vector-borne infectious diseases is anticipated to increase in the coming decades. In endemic areas, rates of malaria and dengue transmission are estimated to rise 12%–27% and 31%–47%, respectively, due to the predicted impacts of climate change [23]. Models designed to forecast future changes in malaria transmission exhibit some variability. However, they consistently predict that the regions currently most vulnerable to malaria are likely to experience increases in populations at risk as a consequence of temperature changes [24, 25]. Additionally, half of the world's population is already at risk for dengue fever [26], and climate change is expected to propagate both geographic spread and seasonal transmission duration [27, 28].

Highly virulent avian influenza viruses are an ongoing public health concern. As of July 2024, a novel highly pathogenic H5N1 strain infected >100 million poultry in 48 US states [28, 29]. A 2006 study analyzing phylogenetic relationships of H5N1 viral isolates to track the spread of avian flu identified the poultry trade rather than migratory birds as the predominant route of introduction in certain regions [30]. In addition, climate change is implicated in altered bird migration patterns, resulting in higher densities of heterogeneous bird populations [31]. Given that heightened interspecies viral transmission can facilitate virus evolution, expansion of agricultural land at the expense of natural habitats is expected to increase interactions between wild and domesticated birds, further enhancing transmission rates [32].

Outside of land use, aquafarming for food production is also correlated to negative health consequences such as a rise in *Vibrio* disease. *Vibrio* spp. are gram-negative bacteria found in the normal microbiota of aquatic animals. Rising water temperatures and changes in salinity in part related to aquafarming are implicated in the increased incidence of pathogenic *Vibrio* disease [33–37]. Other driving factors include aquatic pollution from sewage, crude oil, and industrial run-offs that result in decreased fish diversity and subsequently higher prevalence of pathogenic *Vibrio* disease [38, 39].

## ANTIMICROBIAL RESISTANCE

Antimicrobial resistance is 1 of the top 10 public health threats according to the World Health Organization (WHO) [40]. One factor contributing to this global issue is food production, particularly within the animal agriculture industry. Antibiotics are used in animal feed to improve growth, reproductive performance, and disease prevention [41]. Antibiotic use in food-producing animals between 2010 and 2030 is predicted to nearly double in certain parts of the world to match global demand [41]. Alarmingly, the Natural Resources Defense Council (NRDC) estimates that currently around 65% of antibiotics sold in the United States are designated for animal agriculture, predominantly for cattle and swine [42].

Antibiotic use in animal feed leads to the transfer of the antibiotic and its metabolites from animal manure into soil and runoff [43], with numerous potential downstream effects [44, 45]. About 75% of antibiotics administered in livestock are not absorbed and are ultimately excreted into animal waste [46]. Contaminated manure from animal waste is present in fertilizer and crop irrigation. Subsequent human consumption of such crops acts as a mechanism for introducing bacterial resistance [47]. Studies from countries such as Germany and China detected significant numbers of antibiotic-resistant bacteria in wastewater treatment plants because of high-volume antibiotic use and runoff related to animal farmland [48, 49].

**Figure 1.**
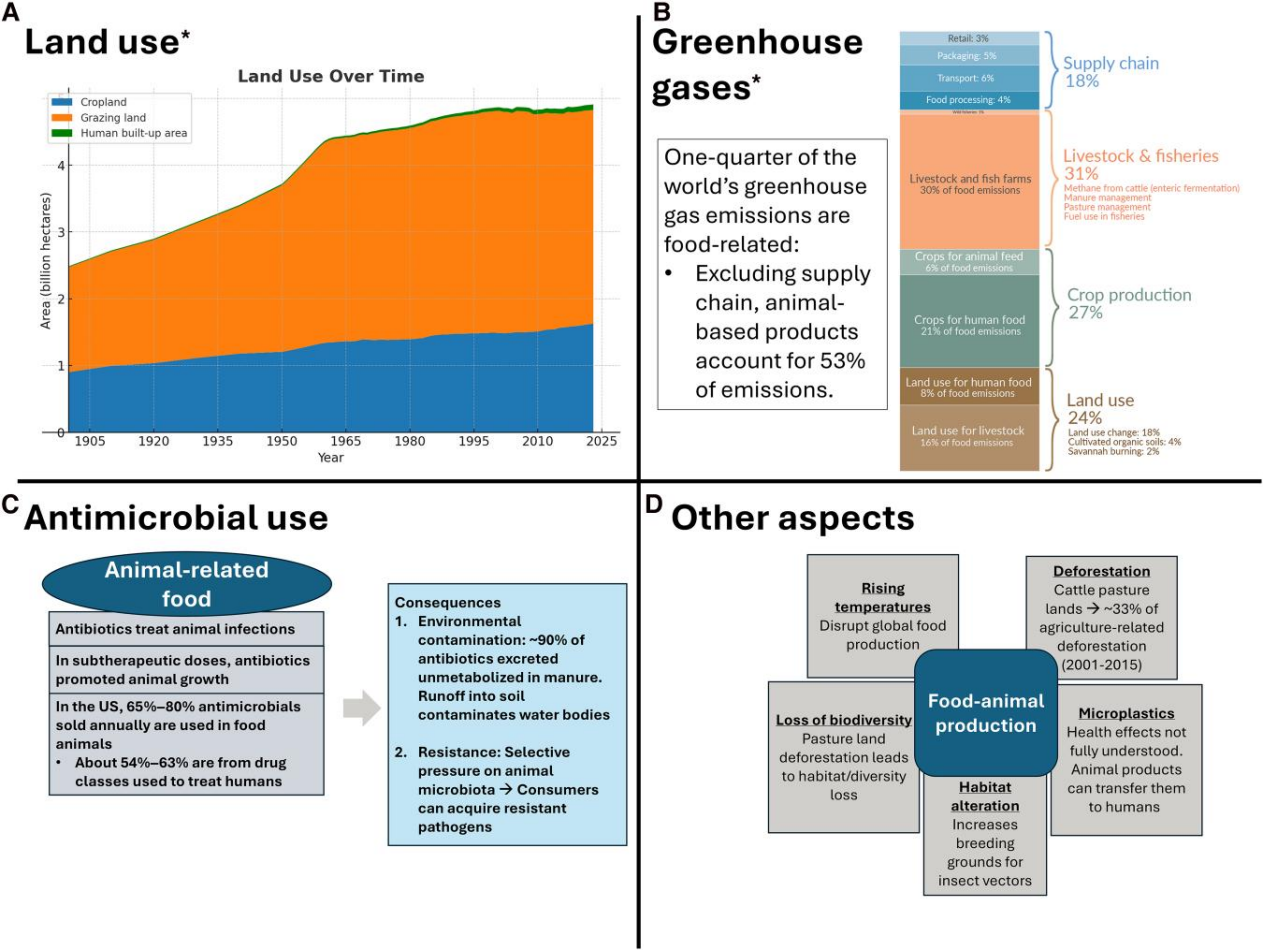
Environmental consequences of dietary animal products. *A,* Graphical reflection of global land use over the past 120 years [11] . *B,* Breakdown of greenhouse gas emissions related to food production [12]. *C,* Consequences of antibiotic use in animal agriculture [51, 52]. *D,* Other negative consequences of animal-based food production. *By authors Hannah Richie and Max Roser licensed under Creative Commons License CC-BY.

In 2021, the Food and Agriculture Organization of the United Nations (UN) updated their detailed action plan on antimicrobial resistance to slow the spread of resistant pathogens within the agriculture sector [50]. In past years, areas such as the European Union and Denmark led successful initiatives to restrict antibiotic use in animal feeds. However, despite recognition of this as an area of concern for decades, few impactful policies exist within the United States [45]. Implementation of the goals outlined by the UN to decrease antimicrobial use in food-producing animals will require global coordination and significant changes in legislature. While large-scale changes are necessary, at an individual level a reduction in demand for animal product consumption can be helpful to facilitate meaningful change (Figure 1).

## IMPACT ON HUMAN HEALTH

Evidence suggests that plant-based diets improve health compared with diets composed of higher amounts of animal-derived products [10]. Well-recognized health benefits of a plant-based diet include reduced risk of cardiovascular disease, obesity, type 2 diabetes mellitus [53, 54], and overall decreased mortality [55]. Health benefits are thought to be in part related to beneficial effects on the immune system. Plant-based foods contain higher amounts of dietary fiber, beta-carotene, vitamins C and E, and polyphenolic flavonoids, all of which are known antioxidants and mediators in immune function [56, 57].

In contrast, high consumption of red and processed meat is associated with negative health consequences. The WHO endorses reducing the intake of red meat and processed meat due to its association with colorectal cancer [58]. Further studies identified additional associations between red meat consumption and other cancer types such as breast cancer and lung cancer [59]. Meat consumption is also associated with other negative health consequences. For example, a large cohort study conducted in the United Kingdom among almost 500 000 adults found that higher meat consumption rates (both processed and unprocessed) were associated with higher rates of ischemic heart disease, digestive diseases, and diabetes [60, 61]. While confounders such as obesity and body adiposity do exist, overall plant-based diets promote healthier body weights as well as overall health benefits. Thus, numerous medical associations and international practice guidelines suggest a plant-based diet as a part of a healthy lifestyle and disease prevention [10, 62].

## THE GASTROINTESTINAL MICROBIOME

The importance of the gut microbiota in the health–disease continuum is increasingly recognized [63]. The gut microbiota has expanded our understanding of food beyond its nutritional value, as both short-term and long-term dietary changes significantly influence its composition and function [64]. Plant-based diets are rich in polyphenols found in fruits and vegetables and interact with gut microbiota in the ileum and colon. Polyphenols promote the abundance of bacteria that confer anti-inflammatory and antipathogenic properties, support intestinal barrier integrity, and enhance lung and central nervous system function [64]. Additionally, the carbohydrate residues in the form of fiber in plant-based diets are metabolized by colonic microbiota, leading to the release of metabolites that regulate colonic mucosal inflammation and proliferation [65]. When fiber intake is high, the metabolic requirements of the colonic mucosa are exceeded, and the surplus of metabolites exerts epigenetic and immunomodulatory effects on other organs [65]. This may explain the association between high dietary fiber intake and decreased mortality, as well as a reduced incidence of conditions such as colon cancer, breast cancer, liver cancer, infections, and respiratory diseases [65].

## OBSERVED CLINICAL BENEFITS

Observational data suggest that plant-based diets may be protective against various infectious diseases [66]. A large community-based prospective cohort study utilized a food frequency questionnaire to assign participants a plant-based diet index (PDI) and subsequently stratified between healthy and unhealthy PDIs based on questionnaire responses. Among individuals who reported a healthy PDI, those in the highest quintile experienced a risk of hospitalization due to respiratory infection that was 16% lower than those in the lowest quintile [67]. Another study conducted during the first year of the coronavirus disease 2019 (COVID-19) pandemic analyzed country-specific trends in fruit and vegetable consumption and found both to be inversely correlated with COVID-19 incidence and death [68]. Obesity, animal products, and animal fats were diet-related factors that correlated with increased COVID-19 incidence and death. In another COVID-19-related case–control study among health care workers in 6 countries, diets that were reported as “plant-based” or “pescatarian” correlated with 73% and 59% lower odds of moderate to severe COVID-19 compared with high-protein, low-carbohydrate diets, respectively [69]. Similarly, a cross-sectional study of 250 hospitalized patients with COVID-19 found associations between higher consumption of fruits, vegetables, and dietary fiber with milder COVID-19 infections and shorter hospitalizations [70].

Persons with HIV (PWH) face twice the risk of cardiovascular disease compared with the general population [71]. Dietary counseling emphasizes that the anti-inflammatory benefits of plant-based diets could help reduce this risk in PWH. The 2019 American Heart Association (AHA) guidelines advocate for primary prevention of cardiovascular disease through lifestyle modifications, which should be particularly emphasized for PWH [72]. Specifically, the AHA recommends a high-fiber plant-based diet due to its association with lower all-cause mortality. Conversely, avoidance of red meat is advised as high intake of animal fat and protein is linked to an increased risk of both cardiac and noncardiac mortality [72]. Besides HIV infection, smaller studies suggest that plant-based diets may reduce the prevalence of primary cancer progression with other chronic viral infections such as hepatitis C and human papillomavirus (HPV) [66, 73, 74]. While these data are mostly observational and limited by small sample sizes, given the low associated risks, dietary modification counseling in these populations still likely results in health benefits.

## APPLICATION TO CLINICAL CARE

The dietary patterns of a substantial portion of the US population are unhealthy, with only about 10% meeting the recommended daily fruit and vegetable intake [75, 76]. Eating habits are complex and arise from an interplay of individual factors (eg, knowledge and food preferences), socio-cultural factors (eg, political aspects, social norms, social support), economic factors (eg, income, food cost), and physical factors (eg, the food environment changes due to food availability) [10, 77]. Health care providers play an important role in dietary counseling, although multiple barriers exist. First, there is no consensus framework for dietary counseling by physicians. Physicians and other health care providers may also feel inadequately trained, as nutrition is an underaddressed topic in medical school and residency training [78, 79]. Other barriers include lack of faculty expertise at residency training sites, lack of reimbursement, and inadequate time in both the hospital and ambulatory settings [78]. While time constraints are a limiting factor, a multidisciplinary approach can help mitigate this barrier. Primary care and registered dieticians should be utilized resources when available. Additionally, counseling may be impractical in the inpatient service given high consultation volumes but may be feasible in the outpatient setting such as a hospital follow-up visit.

**Figure 2.**
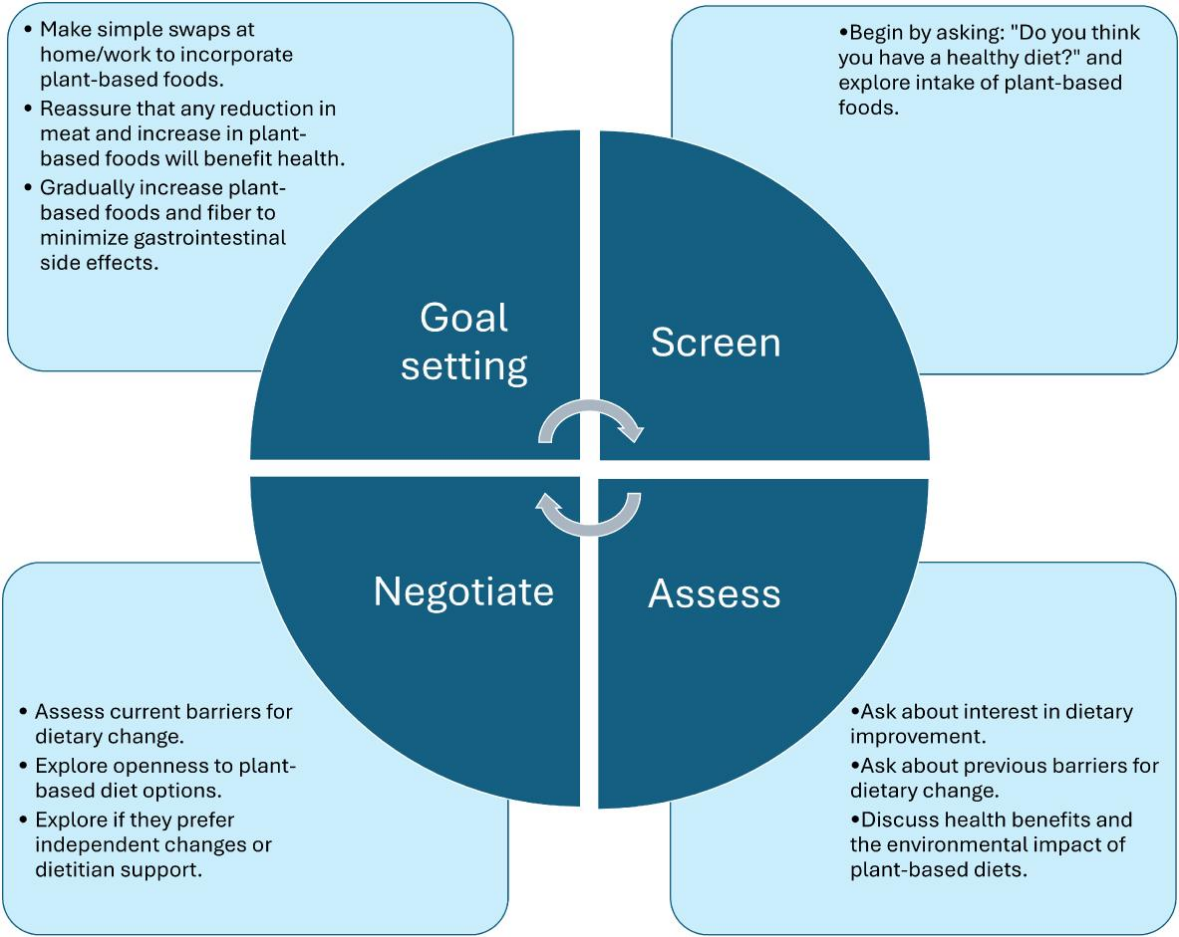
Proposed approach to patient counseling on plant-based diets.

Overall, nutritional counseling as part of primary prevention for numerous chronic diseases and infections is a vital part of effective patient care, as disease prevention remains a goal for generalists as well as infectious disease specialists. A framework to discuss dietary counseling on shifting toward a plant-based diet with patients is shown in Figure 2.

When discussing dietary modifications, it is important to address socioeconomic barriers and how they may affect access to healthy, plant-based food. A cross-sectional study using data from the National Health and Nutrition Examination Survey demonstrated that participants in the lowest poverty index ratio group were most likely to have the lowest consumption of healthier plant-based foods [80]. Food deserts, or areas with limited access to healthy, affordable food, and food swamps, areas with high access to fast food or processed foods, are areas with much higher rates of obesity and obesity-related cancer mortality [81]. Areas of food insecurity and by consequence higher amounts of obesity were disproportionately impacted by the COVID-19 pandemic, with higher rates of infection and severity of illness [82, 83]. Multiple factors are thought to play a role in the development and persistence of food deserts, including lack of incentive for grocery stores to invest in areas of low socioeconomic status and higher fast-food restaurant density in these same areas [84, 85]. Large-scale changes related to health policy are needed to positively impact areas of food insecurity given the influence that the US meat and dairy industry as well as fast-food lobbyists has on lawmakers at present [86, 87].

Fortunately, the availability of healthier plant-based food alternatives is increasing in recent years, fostering an increase in the consumption of plant-based diets. For example, from 2017 to 2023, plant-based food sales doubled from $4 billion to $8 billion, with more plant-based alternatives available on the market than ever before [88]. One of the most frequently cited reasons for why consumers may not choose plant-based alternatives is differences in price. However, data support that this may not always be the case. A 2021 international modeling study using regionally comparable food prices and socioeconomic change scenarios up to 2050 found that compared with current dietary costs more healthy and sustainable dietary patterns were associated with 22%–34% lower costs in higher-income countries such as the United States [89]. A secondary analysis of a randomized controlled trial assessing the effects of vegan diets on overweight adults found that mean total food costs per day in the vegan group decreased by 16%, compared with no significant change in the control group [90]. As more plant-based alternatives come to market and the global meat demand outweighs supply, it is anticipated that costs of plant-based foods will continue to decrease in the coming years.

As leaders in multiple facets of health care systems, infectious disease specialists can shape hospital practices by improving plant-based menu options. In 2023, the largest New York City health system shifted to plant-based foods as the default for inpatient meals, subsequently reducing its food-based carbon emissions by 36% [91]. Provided meals were accepted by 9 out of 10 patients, with an initial cost savings of 59 cents per tray [92]. As leaders in infection control and stewardship, infectious disease providers should promote healthier, plant-based food options in health care systems as a practical and cost-saving change. Resources from pages such as Practice Greenhealth and My Green Doctor offer suggestions on how to initiate some of these changes.

We recognize that a complete switch to plant-based diets is not feasible or culturally acceptable for many patients. This review hopes to encourage counseling on small, gradual changes rather than complete elimination of animal products from a patient's diet. Some suggested changes include “meatless Mondays” or avoidance of animal products before dinnertime. Furthermore, animal-based foods are present in many cultural and religious practices and should be acknowledged. Incremental reductions in animal product consumption should be suggested to help foster broader acceptance of plant-based dietary modifications. Table 2 illustrates additional potential barriers to plant-based diet availability and intake with proposed solutions that may address these barriers.

**Table 2.** Barriers and Possible Solutions to Increase Plant-Based Diet Availability and Intake

Barrier	Potential Solutions
Lack of physician training	Offer free CME courses (NextGen.U, NutritionInMedicine.org, Stanford CME course - Introduction to Food and Health) Education at medical conferences Consider referral to Registered Dietitians, when appropriate and if accessible
Price barriers/access	Advocate for subsidies in plant-based foods Develop urban farms/gardens in hospitals serving food deserts
Social/cultural	Suggest small, practical dietary changes such as “meatless Mondays” or “no meat before dinner” Remind patients that change is small and incremental rather than an “all or nothing” approach Recognize and validate a patient's cultural or religious practices
Concern for adequate nutrient content	Improve patient education on the nutritional profile of a plant-based diet, including dietary protein needs Offer a counseling visit to a Registered Dietician if that resource is available Provide patient-friendly resources (Kaiser Permanente's starter kit, “plant-based eating”)
Taste preference challenges	Discuss that taste preferences relate to previous eating patterns and change with time Expansion of better tasting plant-based alternatives
Lack of exposure to plant-based diets	Increase exposure to healthy, tasty plant-based diets in public spaces (eg, hospitals with plant-focused menus)

Abbreviation: CME, continuing medical education.

## CONCLUSIONS

Climate change is an international crisis, and a multifaceted approach is needed to offset its anticipated impacts on human health. The animal agriculture industry is responsible for greater than 30% of anthropogenic greenhouse gas emissions and the use of over one-third of the world's habitable land [12, 93]. Transitioning to plant-based diets is a sustainable choice that contributes less to climate change while offering significant health benefits compared with diets high in animal-based food products. Even modest reductions in meat consumption can lead to substantial environmental benefits. For instance, if every individual in the United States decreased their meat consumption by 25%, they would achieve a 1% annual reduction in greenhouse gas emissions and liberate land equivalent to the size of Indiana from animal agriculture–related use [94]. Multiple medical organizations now endorse plant-based diets with limited red and processed meat consumption for chronic disease management [51, 95–98]. Considering the benefits for both environmental sustainability and human health, health care providers should incorporate dietary counseling focused on a more plant-based diet into their practice.
